# Laser diode irradiation mitigates salt stress in rice through coordinated physiological and molecular responses

**DOI:** 10.3389/fpls.2025.1653494

**Published:** 2025-09-10

**Authors:** Feng Cheng, Yetong Qi, Kangqi Lei, Han Yang, Yumeng Lei, Temoor Ahmed, Xingjiang Qi, Zhitao Li

**Affiliations:** ^1^ Xianghu Laboratory, Hangzhou, Zhejiang, China; ^2^ Agricultural Technology Extension Center of Zhejiang Province, Hangzhou, Zhejiang, China

**Keywords:** antioxidants, laser diode, salinity stress, rice, ion homeostasis, multi-omics

## Abstract

Soil salinization affects approximately 20% of cultivated land globally, posing significant threats to rice production and food security. Although conventional approaches have been attempted to enhance salt tolerance in rice, several issues have arisen, such as high costs, complexity and application challenges. The potential of laser diode (LD) technology to enhance plant resilience to salinity stress remains underexplored. This study investigated the potential of red-blue LD at a 3:1 ratio and intensities of 5, 10, or 15 μmol m^−2^ s^−1^ PPFD on salt tolerance in rice seedlings using integrated phenotypic, physiological, transcriptomic, and metabolomic analyses. LD-treated seedlings exhibited significantly enhanced growth parameters, including increased plant height, stem diameter, and root morphology as compared with control. Photosynthetic efficiency was substantially improved, with elevated chlorophyll content and enhanced gas exchange parameters. LD treatment maintained ionic homeostasis by reducing Na^+^ accumulation while preserving K^+^ content, resulting in lower Na^+^/K^+^ ratios. Notably, LD treatment at 15 μmol m^−2^ s^−1^ PPFD substantially enhanced the antioxidant enzyme activities such as SOD (63%), POD (62%), CAT (54%), and APX (14%) in rice leaves as compared to control. Correspondingly, oxidative damage markers were significantly reduced, with H_2_O_2_ and MDA levels decreased while proline accumulation increased. Transcriptome sequencing analysis showed that the application of red-blue laser upregulated the expression of genes related to regulatory pathways such as photosynthesis (*OsLhca* and *OsLhcb*), ion homeostasis (*OsNHX*, *OsHKT* and *OsHAK*), and antioxidant defense (*OsSOD*, *OsPOD*, *OsCAT* and *OsAPX*). Metabolomic profiling identified enhanced phenylpropanoid biosynthesis, glutathione metabolism, and flavonoid accumulation as key protective mechanisms. This research demonstrates that red-blue LD irradiation represents a promising sustainable technology for enhancing crop resilience to salinity stress through coordinated physiological and molecular responses.

## 1 Introduction

Rice (*Oryza sativa* L.), a staple crop for over half the world population, is particularly sensitive to salt stress, especially during seedling and reproductive stages (da Silva Alves et al., 2025). Soil salinization is a persistent environmental challenge that threatens global agricultural productivity (Haj-Amor et al., 2022; Omuto et al., 2024). According to the latest FAO report released in 2025, over 1.381 billion hectares of land worldwide are affected by salinization, accounting for 10.7% of the global land area. Approximately 20% of irrigated farmland (about 45 million hectares) is impacted by soil salinization. Salt stress impairs plant growth through multiple mechanisms, including damage to the photosynthetic apparatus, ionic toxicity, oxidative stress, and osmotic imbalance (Sackey et al., 2025). High soil salinity reduces water potential, limiting nutrient uptake (e.g., nitrogen, phosphorus, potassium, and calcium) and causing osmotic stress (Gadelha et al., 2021). Excessive Na^+^ and Cl^−^ accumulation leads to ionic toxicity, disrupting homeostasis and impairing photosynthesis by inducing stomatal closure and cytotoxic Na^+^ buildup (Zahra et al., 2022).

Additionally, plants mitigate the toxicity of reactive oxygen species (ROS) induced by salt stress through activating their antioxidant defense system, which comprises enzymatic antioxidants (superoxide dismutase, SOD; catalase, CAT; peroxidase, POD; ascorbate peroxidase, APX) and non-enzymatic antioxidants (ascorbate, glutathione) (Zhang et al., 2016; Dumanović et al., 2021; Kesawat et al., 2023). Current strategies to enhance rice salt tolerance are often limited by high costs, biological risks, and application challenges (Liang et al., 2024). Therefore, there is an urgent need to develop innovative, sustainable, and environmentally friendly approaches for the management of soil salinization. Light is a critical regulator of plant growth, development, and stress responses, acting as both signaling prompt and energy source. Plants perceive light through photoreceptors (e.g., phytochromes for red light and cryptochromes for blue light) and chloroplasts, which mediate responses to abiotic stresses, including salinity (Roeber et al., 2021).

Red (640–660 nm) and blue (430–460 nm) light wavelengths align with the absorption peaks of photosynthetic pigments, optimizing photosynthesis (Wang et al., 2016). These wavelengths influence CO_2_ assimilation, stomatal conductance, and photoprotection via light-harvesting chlorophyll a/b-binding (Lhc) proteins (Shimazaki et al., 2007; Gao et al., 2018). While light-emitting diodes (LEDs) are widely used in plant cultivation, their broad emission spectra and lower energy efficiency limit their precision (Dutta Gupta and Jatothu, 2013; Ma et al., 2021). Laser diodes (LDs), with narrow spectral outputs (<10 nm), high electrical-to-optical conversion efficiency, and tunable wavelengths, offer a promising alternative. LDs enhance photosynthesis, seed germination, and stress resistance compared to LEDs (Ooi et al., 2016; Nadimi et al., 2022; Li et al., 2025). For example, He-Ne laser irradiation effectively mitigated cadmium-induced oxidative stress by reducing ROS levels while enhancing antioxidant enzyme activities (SOD, POD, CAT, APX) and glutathione metabolism in wheat (Zhu et al., 2022). Similarly, Ouf et al. (2024) reported that low-intensity He-Ne laser and methylene blue enhances maize (*Zea mays* L.) growth and mitigates salt stress by improving IAA production, photosynthetic pigments, and antioxidant activities, offering a sustainable agricultural solution. Previous study has confirmed that red-blue LD irradiation improves rice growth and yield (Qi et al., 2025), but its role in mitigating abiotic stresses like salinity remains underexplored.

The objectives of this study were to investigate the effects of red-blue LD irradiation on salt stress tolerance in rice seedlings using integrated phenotypic, physiological, transcriptomic, and metabolomic analyses. We aimed to determine the optimal red-to-blue light ratio and intensity, evaluate physiological responses including photosynthetic parameters and antioxidant enzyme activities, elucidate molecular mechanisms through differential gene expression analysis, and characterize metabolic reprogramming patterns to identify key regulatory pathways underlying laser-mediated salt stress tolerance for sustainable agricultural applications.

## 2 Materials and methods

### 2.1 Plant materials and experimental design

Rice seeds (cv. ZJZ17) were surface-sterilized with 1% sodium hypochlorite for 5 min, rinsed three times with distilled water, and incubated in darkness at 28°C for 48 h. Uniformly germinated seeds were transferred to hydroponic containers with rice-specific nutrient solution under controlled conditions (14 h/day at 28°C, 10 h/night at 25°C, 70 ± 5% relative humidity) (Liu et al., 2019). White LED lamps provided a photosynthetic photon flux density (PPFD) of 150 μmol m^−2^ s^−1^. Laser diodes (LDs; Hangzhou Canruo Xingchen Intelligent Co., Ltd.) with adjustable light quality and intensity supplemented the background illumination. Seeds with consistent germination were sown and placed in a white light incubator with the addition of different laser treatments cultured until the seedlings reached the three-leaf stage (15-days-old seedlings). Thereafter, the seedlings were placed in an incubator with white light only and salt stress was applied with 180 mM NaCl. To determine the optimal red-to-blue light ratio, seedlings were exposed to six treatments: natural light (CK), red LD (R, 660 nm), blue LD (B, 450 nm), red:blue ratios of 3:1 (R3B1), 1:1 (R1B1), and 1:3 (R1B3). Following identification of the optimal ratio (R3B1), seedlings were pretreated with LD for 15 days until the three-leaf stage prior to salt treatment, with light intensities of 5, 10, or 15 μmol m^−2^ s^−1^ PPFD (Qi et al., 2025). After 3 days of salt stress (180 mM NaCl), samples were collected for analysis. Salt stress continued for 4 additional days (7 days total), followed by 14 days of recovery in normal rice nutrient solution without salt. Phenological parameters (plant height, stem thickness, root length, fresh weight, dry weight, root surface area, and root tip count) were measured for 30 randomly selected plants per treatment. Root activity was assessed using a root activity assay kit.

### 2.2 Determination of photosynthesis and gas exchange parameters

Fresh leaf tissue (10 mg) was ground in 1 mL of 95% ethanol, macerated for 30 min, and centrifuged. Absorbance of the supernatant was measured at 665 nm, 649 nm, and 470 nm using a microplate reader to quantify chlorophyll a, chlorophyll b, and carotenoids. Photosynthetic parameters, including net photosynthetic rate (Pn), transpiration rate (Tr), intercellular CO_2_ concentration (Ci), and stomatal conductance (Gs), were measured using the LI-6800 portable photosynthesis system (LI-COR, Lincoln, NE, USA) according to our previous study (Ahmed et al., 2024). SPAD values were determined using a SPAD-502PLUS (Konica, Japan). Measurements were conducted on 10 plants per treatment, with three replicates per plant (Adil et al., 2020).

### 2.3 Determination of ionic content

Samples were rinsed with deionized water, de-greened at 105°C for 30 min, and dried at 70°C to constant weight. For elemental analysis, 0.03 g of ground sample was digested in 6 mL of mixed acid (HNO_3_:HClO_4_, 4:1 v/v) using a temperature gradient (60°C for 1 h, 120°C for 1 h, 150°C for 1 h, and 190°C until white fumes appeared). The digest was analyzed for P, K, Na, Ca, Mg, and Zn using Inductively Coupled Plasma Atomic Emission Spectrometry (ICP-AES) (Adil et al., 2020). Total nitrogen was quantified by digesting 0.05 g of sample in 5 mL of H_2_SO_4_ at 190°C for 30 min, followed by 5 mL of 30% H_2_O_2_ at 280°C for 30 min. The clarified digest was filtered (0.45 μm) and analyzed using a continuous flow analyzer (AA3, Seal Analytical, Norderstedt, Germany).

### 2.4 Determination of antioxidant enzymes and ROS activity

Leaf and root tissues (0.1 g per treatment) were homogenized in liquid nitrogen, centrifuged, and analyzed for antioxidant enzyme activities (SOD, POD, CAT, APX) using assay kits (Grace Biotechnology, Suzhou, China). ROS accumulation was assessed by measuring malondialdehyde (MDA) and hydrogen peroxide (H_2_O_2_) levels with colorimetric assay kits (Grace Instrument Biotechnology, Suzhou, China). Antioxidant enzyme activities, MDA and H_2_O_2_ contents were obtained according to the mass of the samples following the calculations in the kit. Proline content was quantified using a proline assay kit (Boxbio, Beijing, China). H_2_O_2_ was visualized using 3,3’-diaminobenzidine (DAB) staining, followed by chlorophyll removal with 95% ethanol at 80°C and fixation for imaging.

### 2.5 Transcriptomic analysis

Leaf tissues from seedlings at the three-leaf stage, treated with LD (5, 10, or 15 μmol m^−2^ s^−1^ PPFD) and subjected to 3 days of salt stress, were collected for transcriptome analysis. Each treatment was sampled as a mixed group of 10 rice seedlings as a biological replication, and each treatment was subjected to three sets of biological replications. Total RNA was extracted using the RNAprep Pure Plant Kit (Tiangen Biotech, Beijing, China), and libraries were sequenced on the Illumina NovaSeq X Plus (Novogene, Shanghai, China). Clean reads were mapped to the *Oryza sativa* Japonica Group reference genome (IRGSP-1.0) using HISAT2 v2.2.1. Gene expression was quantified as Fragments Per Kilobase of transcript per Million mapped reads (FPKM) using StringTie v2.1.4. Differentially expressed genes (DEGs) were identified with |log_2_(fold change)| ≥ 1 and adjusted p-value ≤ 0.05 (Benjamini-Hochberg correction) (Robinson et al., 2010). Gene Ontology (GO) and KEGG pathway enrichment analyses were performed using clusterProfiler v4.0 (padj < 0.05) (Qi et al., 2025).

### 2.6 Metabolomics analysis

Rice seedlings were irradiated with laser LD (15 μmol m^−2^ s^−1^ PPFD) until the three-leaf stage and then subjected to salt stress treatment (180 mM NaCl) for 3 days. Non-LD treated seedlings served as controls. Each treatment was sampled in a mixed group of 10 rice seedlings as a biological replication, and each treatment was subjected to six sets of biological replications. Leaf samples from each treatment group were collected and analyzed using untargeted metabolomics via liquid chromatography-tandem mass spectrometry (LC-MS/MS). Metabolite extraction involved grinding an appropriate sample mass in a suitable volume of extraction solvent, followed by ultrasonication. The mixture was allowed to stand, centrifuged, and the supernatant was vacuum-dried (Want et al., 2006). The residue was reconstituted in an appropriate volume of extraction solvent for LC-MS/MS analysis. Raw data, acquired using MassLynx V4.2, were processed with Progenesis QI software (Guo et al., 2024). Statistical analysis, metabolite classification, annotation, and functional interpretation were conducted on the BMKCloud platform (www.biocloud.net). Six biological replicates were included for each treatment.

### 2.7 qRT-PCR validation

Total RNA from samples used for transcriptome sequencing was extracted according to the RNA extraction kit manufacturer’s instructions. First-strand cDNA was synthesized by the Prime Script™ RT kit (TaKaRa) and used as a template for expression assays. A SYBR^®^Premix Ex Taq™ II (TaKaRa) with CFX96 Real-Time PCR Detection System (Bio-Rad) was used to perform qRT-PCR to check the expression of the genes of selected. The rice Ubiquitin gene was used as an internal reference and relative transcriptional level was calculated using the 2^-ΔΔCt^ method. Three biological replicates and three technical replicates were performed for each sample. The primer sequences were listed in **Supplementary Table S1**.

### 2.8 Statistical analysis

Data are presented as mean ± standard error of the mean (SEM). For pairwise comparisons between two datasets, student’s t-test was employed. For comparisons involving three or more datasets, one-way analysis of variance (ANOVA) was performed using Tukey’s HSD test. One-way ANOVA followed by Duncan’s multiple range test was performed using GraphPad Prism 8.0 (GraphPad Software, San Diego, USA) and Statistix 8.1 (Analytical Software, Tallahassee, USA). Statistical significance was set at *p* < 0.05.

## 3 Results

### 3.1 Effects of laser treatment on rice growth parameters

**Figure 1:**
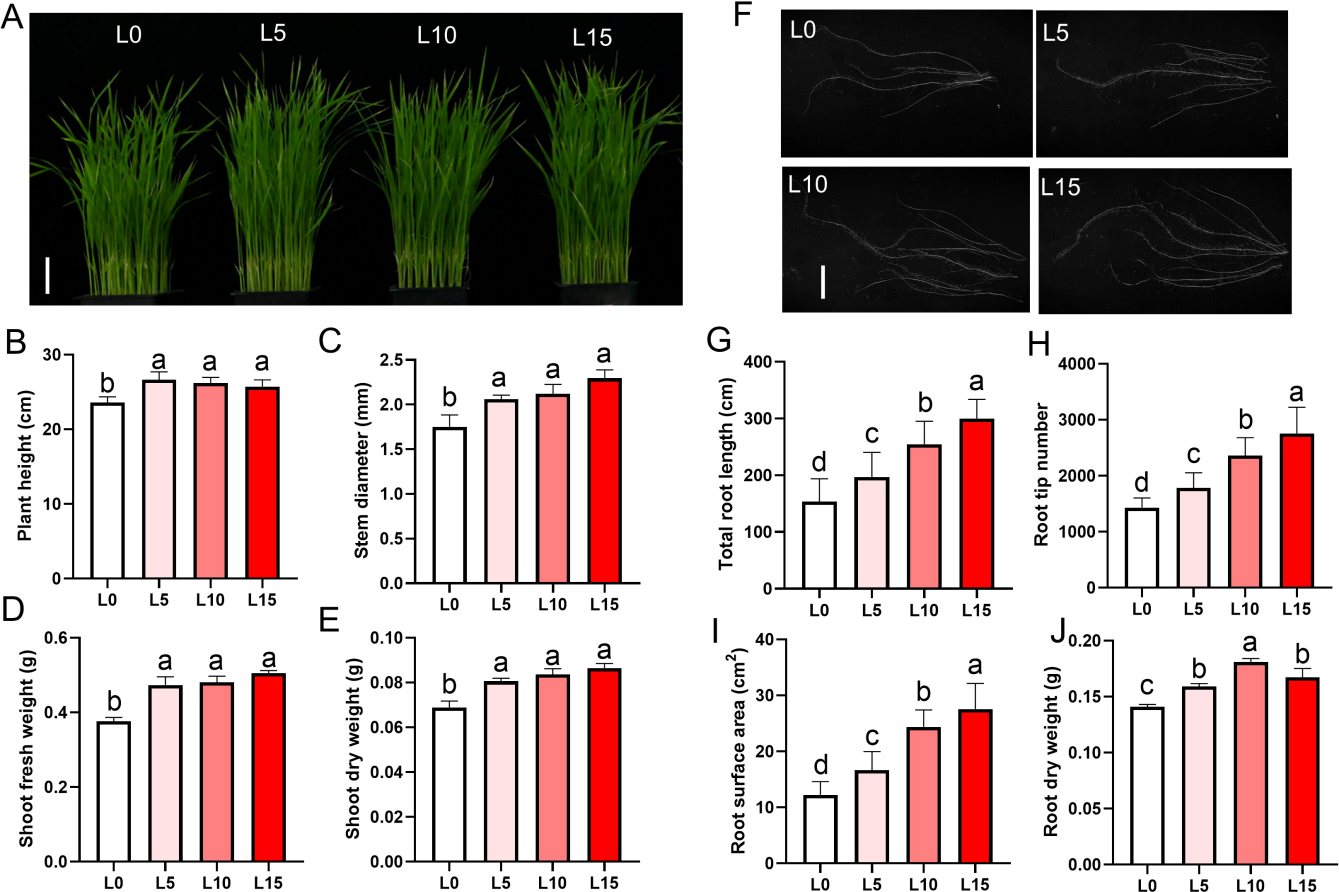
Laser diode irradiation improves growth and root development of rice seedlings **(A)** Representative phenotypes of whole seedlings. Scale bar, 5 cm. **(B–E)** Quantitative analysis of shoot parameters: Plant height **(B)**, stem diameter **(C)**, shoot fresh weight (FW) **(D)**, and shoot dry weight (DW) **(E)**. Scale bar, 5 cm. **(F)** Root system architecture. Scale bar, 1 cm. **(G–J)** Quantitative analysis of root traits: total root length**(G)**, root tip number**(H)**, root surface area **(I)**, and root DW **(J)**. L0, L5, L10, and L15 denote laser intensities at 0, 5, 10, and 15 μmol m^−2^ s^−1^ PPFD, respectively. The data are presented as the mean ± SE, n = 30. Letters above bars indicate statistical significance.

**Figure 2:**
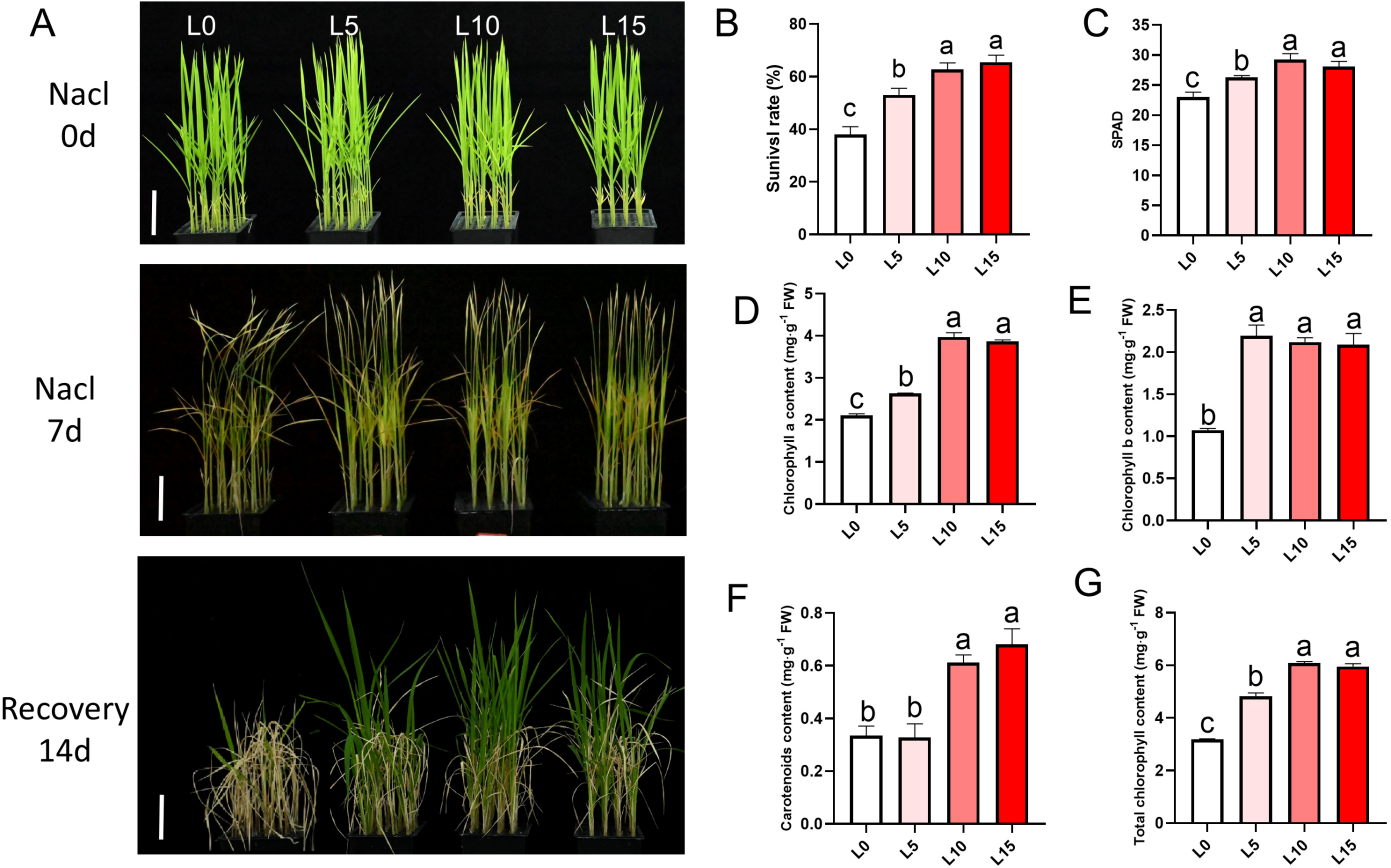
Laser diode treatment improves survival, chlorophyll content, and recovery of rice seedlings under salt stress. **(A)** Phenotype showing salt tolerance detection of rice seedlings after treatment with different intensities. Scale bar, 5 cm. **(B)** Statistics of survival rates after 14 days of recovery treatment. **(C)** SPAD values of rice seedlings under different treatments after 3 days of salt stress. **(D–G)** Photosynthetic pigment contents in rice seedlings under different treatments: chlorophyll a **(D)**, chlorophyll b **(E)**, carotenoids **(F)**, and total chlorophyll content **(G)**. Letters above bars indicate statistical significance.

Phenotypic assessments were conducted on rice seedlings irradiated with LD until the three-leaf stage and subjected to 3 days of salt stress (**Supplementary Figure S1A**). Using the optimal R3B1 ratio, seedlings were exposed to red-blue LD at intensities of 5, 10, or 15 μmol m^−2^ s^−1^ PPFD (designated L5, L10, and L15, respectively) to evaluate salt tolerance. Compared to the non-irradiated control (L0), the L5, L10, and L15 groups showed significant increases in plant height, stem diameter, fresh weight, and dry weight (**Figures 1A–E**). Root morphology was also enhanced in LD-treated groups, with longer primary roots, greater total root length, larger root surface area, higher root tip counts, and increased root dry weight, particularly in the L10 and L15 groups (**Figures 1F–J**). After prolonged salt stress (7 days), control seedlings (L0) exhibited wilting and severe damage, whereas LD-treated seedlings (L5, L10, L15) displayed greater resilience, with more pronounced recovery after 7 days in normal rice nutrient solution (**Figure 2A**). Survival rates were significantly higher in LD-treated groups compared to L0, with L10 and L15 outperforming L5 (**Figure 2B**). These results show that laser treatment promotes both shoot and root development of rice seedlings, especially at intensities of 10 or 15 μmol m^-^² s^-^¹ PPFD.

### 3.2 Effect of laser treatment on rice photosynthesis profile

Rice seedlings treated with a 3:1 red-to-blue light ratio showed significantly higher chlorophyll content compared to other ratios (**Supplementary Figures S1B–D**). Survival rates after 7 days of recovery in normal rice nutrient solution further indicated that the R3B1 ratio conferred superior salt tolerance (**Supplementary Figure S1E**). Additionally, LD-treated seedlings retained greener leaves, as confirmed by higher SPAD values after 7 days of salt stress (**Figure 2C**). Photosynthetic pigment analysis revealed elevated levels of chlorophyll a, chlorophyll b, carotenoids, and total chlorophyll in the L5, L10, and L15 groups compared to L0 (**Figures 2D–G**), underscoring the protective effect of LD irradiation on photosynthetic capacity under salt stress.

**Figure 3:**
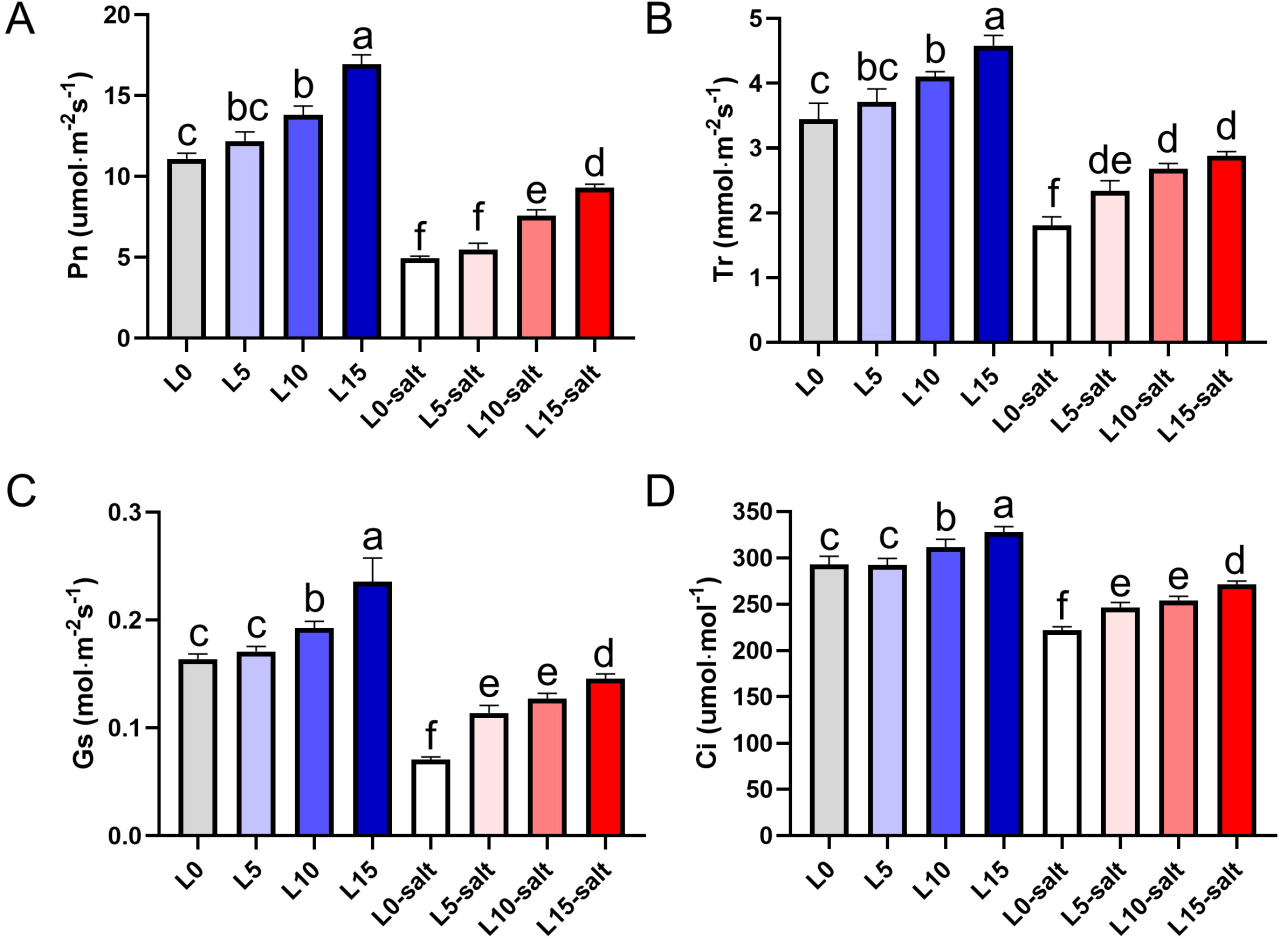
Laser diode treatment improves photosynthetic parameters in rice seedlings under normal and salt stress conditions. **(A)** Net photosynthetic rate (Pn); **(B)** Transpiration rate (Tr); **(C)** Intercellular CO_2_ concentration (Ci); **(D)** Stomatal conductance (Gs). Letters above bars indicate statistical significance.

Photosynthetic results demonstrated that LD treatments significantly improved Pn, Tr, Ci, and Gs in rice seedlings under salt stress (**Figure 3**). Salt stress significantly reduced Pn in the non-LD treated group (L0), but LD treatments at 5, 10, and 15 μmol m^−2^ s^−1^ PPFD (L5, L10, and L15, respectively) significantly mitigated this decline (**Figure 3A**). Compared to L0, Tr increased by 29.6%, 40.5%, and 50.4% in L5, L10, and L15, respectively (**Figure 3B**). Similarly, Ci rose by 12.2%, 15.7%, and 23.5% in L5, L10, and L15 compared to L0 (**Figure 3C**). Although salt stress reduced Gs relative to non-stressed conditions, LD-treated groups exhibited higher Gs than L0 under salt stress, with the effect being more pronounced at higher intensities (**Figure 3D**). These findings indicate that LD irradiation enhances photosynthetic performance under salt stress, facilitating partial recovery of seedling growth by improving CO_2_ assimilation and stomatal function.

In summary, LD partially mitigated the damage of salt stress on rice seedlings by increasing the chlorophyll and carotenoid contents and enhancing the photosynthetic efficiency.

### 3.3 Effect of laser treatment on rice ionic homeostasis

**Figure 4:**
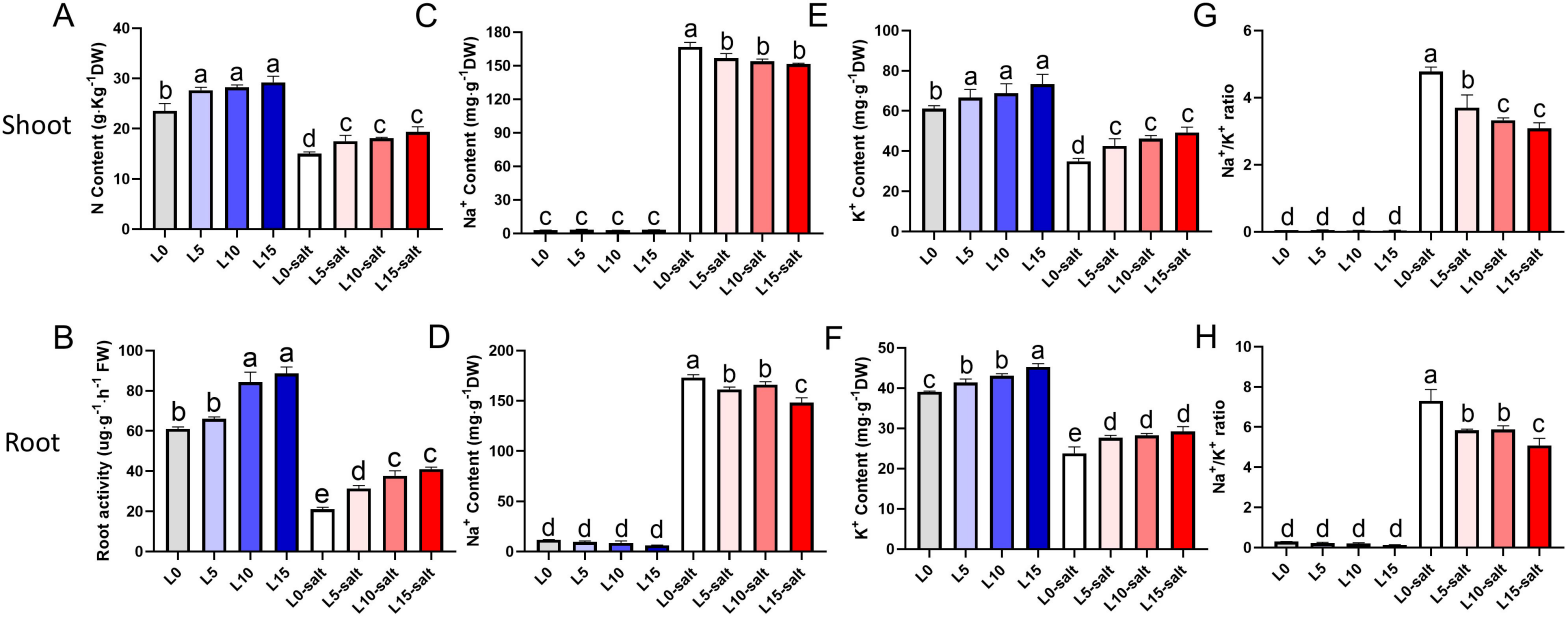
Laser diode treatment effects on ion contents in rice seedlings under normal and salt stress condition. **(A)** Leaf N content. **(B)** Root activity. Na^+^ content in leaves **(C)** and roots **(D)**; K^+^ content in leaves **(E)** and roots **(F)**; Na^+^/K^+^ ratio in leaves **(G)** and roots **(H)**. Letters above bars indicate statistical significance.

These results revealed that N content in the leaves increased in the L5, L10, and L15 groups relative to the L0, suggesting that the laser treatment restored some of the salt stress-induced reduction in N content (**Figure 4A**). Similarly, root activity assays demonstrated enhanced root vigor in LD-treated groups, with laser treatment partially restoring the decline in root activity caused by salt stress (**Figure 4B**). These phenomena indicate that LD treatment enhanced salt tolerance in rice seedlings to a certain extent. The ratio of Na^+^/K^+^ content in plant tissues is another crucial indicator of salt tolerance in crops. Salt stress significantly increased Na^+^ levels and decreased K^+^ levels in rice seedling leaves and roots compared to normal conditions. However, laser treatment reduced this Na^+^ accumulation and restored K^+^ levels under stress relative to the control (L0) group (**Figures 4C–F**). Consequently, salt stress markedly raised the Na^+^/K^+^ ratio, while laser treatment significantly mitigated this increase (**Figures 4G, H**). A high pH environment disrupts the balance of mineral ion uptake in plants. Compared to the L0 group, the P content in the shoot of the L5, L10, and L15 groups increased by 14%, 30%, and 35%, respectively (**Supplementary Figure S2A**). Relative to the L0 group, the L5, L10, and L15 groups showed P content in the roots of the increases of 9%, 18%, and 30%, respectively (**Supplementary Figure S2B**). Statistical analysis further indicated that laser treatment effectively restored the absorption of essential elements, including Ca, Mg, Zn, and Mn, in both shoots and roots of rice seedlings under salt stress (**Supplementary Figures S2C–J**).

In conclusion, the laser balanced ion homeostasis by reducing Na^+^ accumulation and elevating K^+^ content in rice seedlings under salt stress, while enhancing root vigor and mineral element uptake to effectively mitigate ion toxicity.

### 3.4 Effect of laser treatment on antioxidants and ROS activity

**Figure 5:**
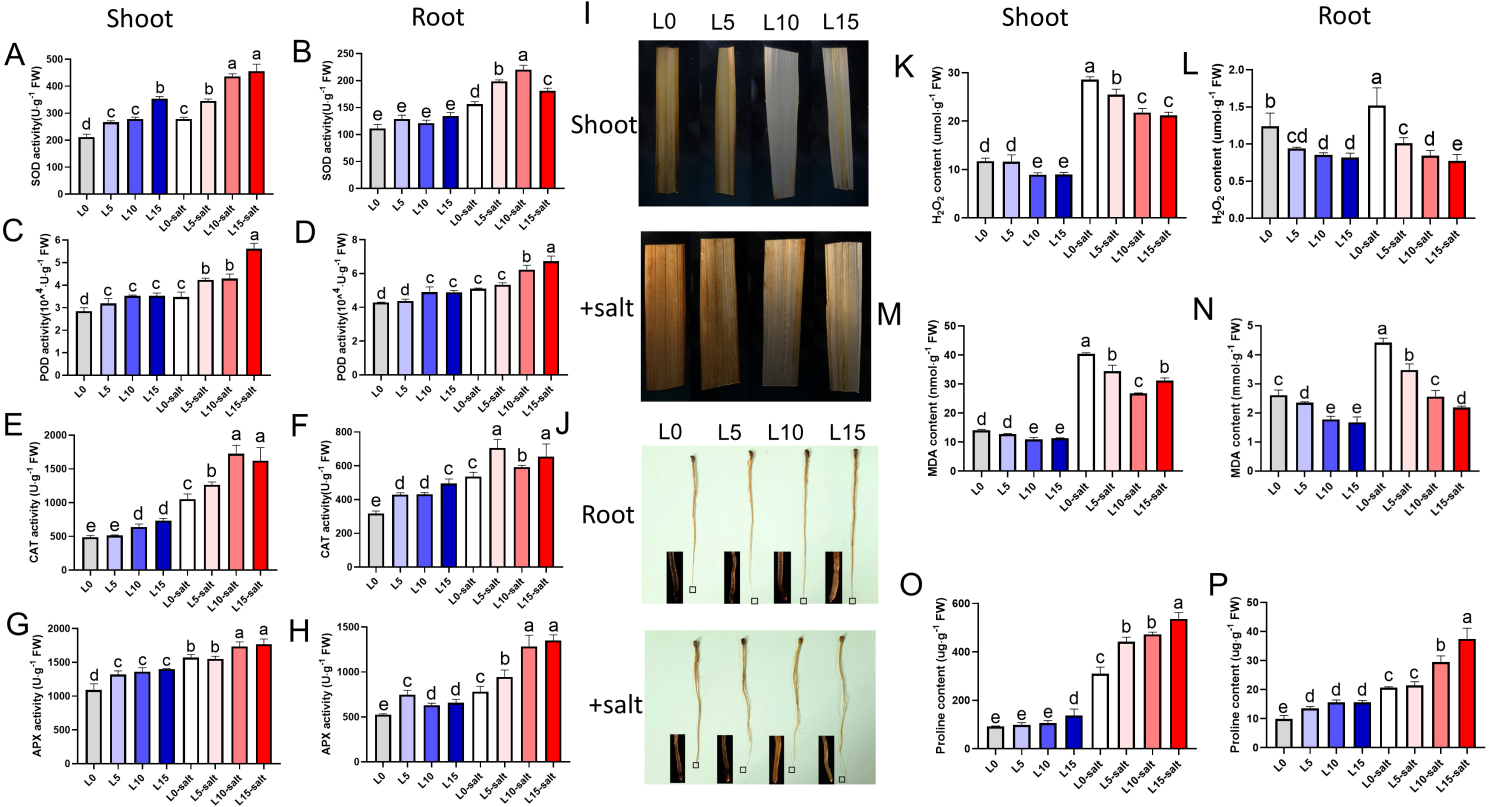
Laser diode treatment effects on antioxidant enzymes and reactive oxygen species (ROS) in rice seedlings under normal and salt stress conditions. **(A-J)** Antioxidant activities: **(A)** SOD activity in leaves. **(B)** SOD activity in roots. **(C)** POD activity in leaves. **(D)** POD activity in roots. **(E)** CAT activity in leaves. **(F)** CAT activity in roots. **(G)** APX activity in leaves. **(H)** APX activity in roots. **(I)** DAB staining for visualizing H_2_O_2_ accumulation in leaves. Scale bar, 1 cm. **(J)** DAB staining for visualizing H_2_O_2_ accumulation in roots. Scale bar, 1 cm. **(K–P)** Stress response markers: **(K)** H_2_O_2_ content in leaves. **(L)** H_2_O_2_ content in roots. **(M)** MDA content in leaves. **(N)** MDA content in roots. **(O)** Proline content in leaves. **(P)** Proline content in roots. Letters above bars indicate statistical significance.

A substantial reduction in antioxidant enzyme activity was noticed in rice plants under salt induced stress condition. However, laser treatment enhanced the activities of antioxidant enzymes in leaves and roots under salt stress conditions. The leaves of L5, L10, and L15 pretreated rice seedlings showed significantly improved SOD activity of 24%, 56%, and 63% compared with the L0 control group, while root SOD activity was significantly improved by 27%, 41%, and 16% compared with the L0, respectively (**Figures 5A, B**). In addition, after salt stress, the POD activity of seedlings in the laser treatment groups increased by 22%, 24%, and 62%, respectively, compared with the L0, and the POD activity of the root system increased by 6%, 22%, and 32%, respectively (**Figures 5C, D**). The CAT activity of seedling leaves in the L5, L10, and L15 laser pretreatment groups was 20%, 63%, and 54% higher than that in the L0, while the CAT activity of the root system was 31%, 10%, and 22% higher than that in the L0, respectively (**Figures 5E, F**). Under salt stress, the APX activity in the leaves of the laser-treated groups increased by 3%, 12%, and 14%, respectively, while the APX activity in the root system increased by 21%, 64%, and 72% compared with the L0 (**Figures 5G, H**).

Salt stress typically induces oxidative damage in rice, leading to the accumulation of reactive ROS. To determine whether elevated ROS levels corresponded to increased cellular damage, we measured H_2_O_2_ and MDA contents across treatment groups. Under normal conditions, laser pretreatment had no significant effect on H_2_O_2_ levels in leaves or roots (**Figure 5I**). However, after salt treatment, laser-pretreated seedlings displayed lighter 3,3’-diaminobenzidine (DAB) staining in both tissues, indicating reduced oxidative damage compared to non-laser-treated controls (**Figure 5J**). The quantitative test results for H_2_O_2_ were consistent with the staining observations (**Figures 5K, L**). LD pretreatment significantly reduced the MDA content in rice seedling leaves and roots under salt stress (**Figures 5M, N**). Notably, LD pretreatment also significantly increased proline levels in rice seedlings (**Figures 5O, P**). These findings demonstrate that LD treatment reduces MDA content and H_2_O_2_ accumulation while increasing activity of key antioxidant enzymes, thus significantly enhancing the cellular protection mechanisms and antioxidant stress capacity of rice seedlings.

In conclusion, laser treatment increased antioxidant enzyme activities and proline content and decreased MDA and H_2_O_2_ accumulation in rice seedlings under salt stress. Therefore, laser may support higher cytoprotective and antioxidant tolerance capacity of rice under salt stress.

### 3.5 Laser treatment regulated transcriptomic changes in rice

**Figure 6:**
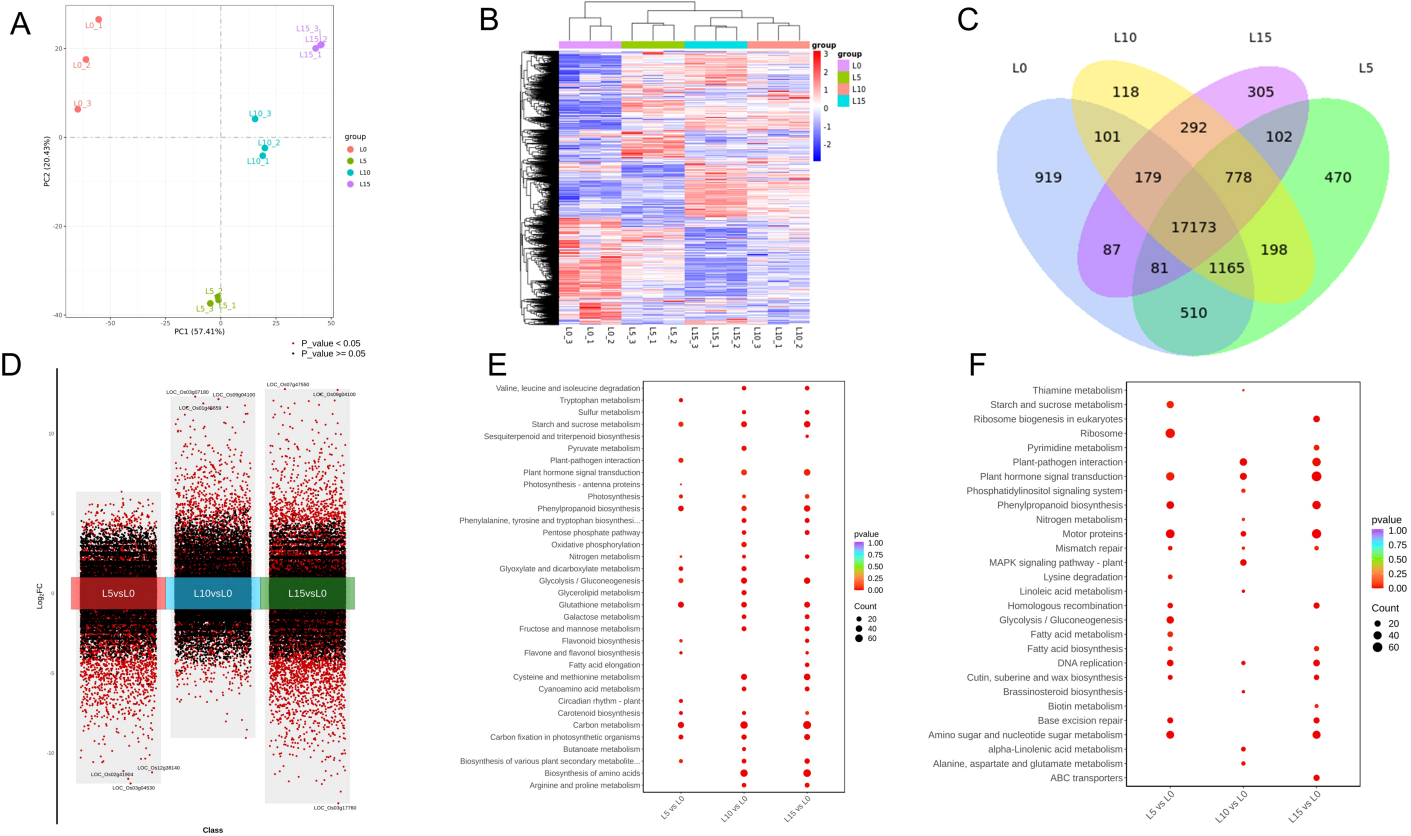
Laser diode treatment regulating transcriptomic profiling of differentially expressed genes (DEGs) in rice under normal and salt stress conditions. **(A)** PCA plot of DEGs across different comparisons. **(B)** Heatmap of DEG expression patterns in distinct clusters. **(C)** Venn diagram analysis of DEGs between comparative groups. **(D)** Statistics of up- and down-regulated DEGs across treatment groups. **(E)** KEGG enrichment of up-regulated DEGs. **(F)** KEGG enrichment of down-regulated DEGs.

To explore the molecular mechanism of laser pretreatment in improving the salt tolerance of rice seedlings, this study conducted transcriptome sequencing analysis on rice leaves in different laser pretreatment groups (L5, L10, L15) and untreated control group (L0) under salt stress. The results of PCA and heatmap of DEG expression patterns showed that laser pretreatment of different intensities significantly affected the transcription level of rice seedlings under salt stress (**Figures 6A, B**). The Venn diagram shows that there are 17,173 differentially expressed genes (DEGs) shared between the L5, L10, and L15 groups (**Figure 6C**). Statistical analysis of DEGs revealed that the number of DEGs increased with higher laser pretreatment intensities, with a particularly notable rise in the number of upregulated genes (**Figure 6D**). Gene Ontology analysis indicated that the upregulated DEGs were primarily enriched in terms related to photosynthesis, photosystem II, oxidoreductase activity, carbohydrate metabolic process, and ion transport (**Supplementary Figure S3A**), whereas the downregulated DEGs were mainly associated with microtubule binding, cellular carbohydrate metabolic process, response to stimulus, and tubulin binding (**Supplementary Figure S3B**). KEGG pathway analysis showed that the upregulated DEGs in the L5, L10, and L15 groups were predominantly involved in carbon fixation in photosynthetic organisms, photosynthesis, starch and sucrose metabolism, carbon metabolism, nitrogen metabolism, carotenoid biosynthesis, flavonoid biosynthesis, glutathione metabolism, and phenylpropanoid biosynthesis (**Figure 6E**). In contrast, the downregulated DEGs were primarily enriched in plant-pathogen interaction, motor proteins, phenylpropanoid biosynthesis, fatty acid metabolism, plant hormone signal transduction, and amino sugar and nucleotide sugar metabolism (**Figure 6F**). To elucidate the effects of red and blue LD pretreatment on chloroplast function in rice seedlings, this study focused on analyzing the expression patterns of photosystem- and chloroplast-related DEGs. The results showed that the expression levels of multiple photosynthesis-related genes in the L5, L10, and L15 groups were significantly higher than those in the L0 group under salt stress. These genes included the photosystem I gene *OsPsaO* and Photosystem II polypeptide genes *OsPsbR1/2/3*, *OsPsbS1/2* and *OsPsbP*. Additionally, the expression of genes related to the light-trapping complexes of PSI and PSII was significantly elevated, including the light-harvesting-like proteins ONE-HELIX PROTEIN1 (OHP1) and OHP2) associated with the stabilization of photosystem II, the light-harvesting chlorophyll a/binding proteins Lhca1-6 associated with photosystem I (PSI), and the photosystem II (PSII)-associated protein Lhcb1-7 (**Supplementary Figure S4A**). Given that photosynthetic pigments are upstream components of light signal transduction, this study further analyzed the expression of photoreceptor-related genes. Heatmap analysis revealed that RBLD pretreatment upregulated the expression of cryptochrome genes (*OsCRY1*, *OsCRY2*, *OsCRY3*), which perceive blue light, while downregulating the expression of phytochrome genes (*OsPhyA*, *OsPhyB*, *OsPhyC*), which perceive red/far-red light. Concurrently, the expression of phytochrome-interacting factor-like (PIL) genes (*OsPIL12*, *OsPIL13*, *OsPIL14*) was upregulated (**Supplementary Figure S4B**).

Because the scavenging of ROS is an important mechanism for plants to withstand oxidative stress and improve salt tolerance, the differences in the expression of genes related to ROS scavenging enzymes were analyzed after laser treatment under salt stress. Key genes in the salt overly sensitive (SOS) pathway were upregulated in the L5, L10 and L15 groups, including *OsSOS1*/*OsNHX7* (Sodium/hydrogen exchanger 7), *OsSOS2*/*OsCIPK24* (CBL-interacting protein kinase 24) and *OsSOS3*/*OsCBL4* (Calcineurin B-like protein 4). Additionally, most of the key genes regulating the absorption, transport, excretion, and distribution of Na^+^ and K^+^ under salt stress were significantly upregulated in the L5, L10, and L15 groups, such as vacuolar membrane Na^+^/H^+^ antiporter genes (*OsNHX1*, *OsNHX2*, *OsNHX3*, *OsNHX5*), high-affinity K^+^ transporter genes (*OsHAK*), and plasma membrane Na^+^/K^+^ transporter-related genes (*OsHKT1*, *OsHKT2*), indicating that LD preconditioning contributed to the maintenance of salt-stressed ionic homeostasis in rice seedlings under salt stress (**Supplementary Figure S4C**). To investigate the response of the antioxidant system under salt stress, the expression of genes related to ROS scavenging enzymes was analyzed. The results showed that after laser pretreatment, the expression of most antioxidants genes such as *OsSODC*, *OsPER*, *OsAPX* and *OsCAT* were significantly upregulated (**Supplementary Figure S4D**). In addition, qRT-PCR results showed that laser treatment under salt stress resulted in elevated expression levels of most photosynthesis-related, salt-response-related, and antioxidant-activity-related genes, which is consistent with the results of the transcriptome data (**Supplementary Figure S5**). In summary, laser pretreatment may synergistically enhance the tolerance of rice seedlings to salt stress by activating photosynthesis-related genes, ion homeostasis regulatory genes, and up-regulating the expression of antioxidant enzyme system genes.

### 3.6 Laser treatment reshaped the rice metabolites

**Figure 7:**
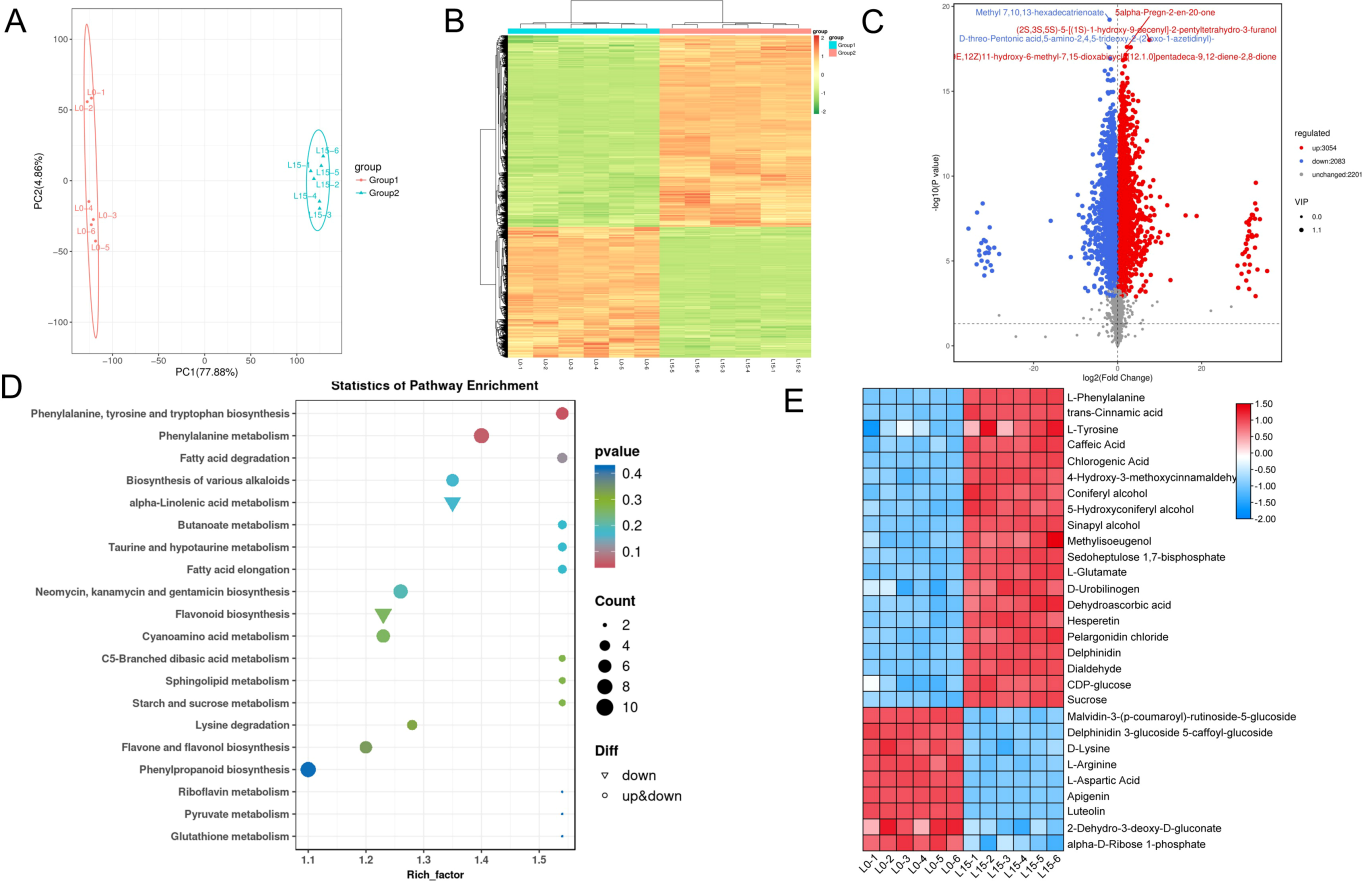
Laser diode treatment regulating metabolites profiling of differentially expressed metabolites (DAMs) in rice under normal and salt stress conditions. **(A)** PCA plot of overall sample analysis. Each point corresponds to a biological replicate; identically colored points denote samples within the same cluster. **(B)** Hierarchical clustering heatmap of DAMs. The horizontal axis represents sample descriptions, the vertical axis displays metabolite profiles, and various colors indicate values derived after normalization of relative content (red indicates high content, green indicates low content). **(C)** Volcano plot of DAMs. Significantly downregulated, upregulated, and non-significant metabolites are shown in blue, red, and gray, respectively. The top 5 annotated metabolites ranked by p-value are labeled. **(D)** KEGG enrichment scatter plot of DAMs. **(E)** Relative abundance of key DAMs. Metabolite levels are color-scaled (red: upregulated; blue: downregulated) relative to the control.

Based on salt tolerance assays and transcriptome analysis, which revealed that rice seedlings under salt stress exhibited enhanced salt tolerance and more pronounced differential responses in L15 compared to L5 and L10, we conducted a metabolomic study using LC-MS to investigate the effects of laser treatment on salt stress tolerance. This study compared seedlings from the laser-treated group (L15) and the control group (L0) under salt stress conditions. PCA revealed distinct metabolic profiles between groups, confirming the reproducibility among biological replicates of L0 and L15 (**Figure 7A**). To effectively visualize patterns of relative metabolite abundance changes, we plotted hierarchical clustered heat maps, demonstrating significant metabolic alterations in the laser-treated group under salt stress (**Figure 7B**). Compared with L0, the levels of 3054 metabolites were elevated, whereas the levels of 2083 metabolites were reduced in rice seedlings from the L15 treatment group (**Figure 7C**). KEGG pathway enrichment analysis indicated that differentially abundant metabolites (DAMs) were significantly enriched in phenylalanine, tyrosine and tryptophan biosynthesis, phenylalanine metabolism, flavonoid biosynthesis, phenylpropanoid biosynthesis, glutathione metabolism, and starch and sucrose metabolism pathways (**Figure 7D**). To identify the key metabolites that laser regulate the tolerance of rice seedlings to salt stress, we performed expression profiling of differential metabolites of key pathways. Heatmap analysis revealed significantly increased abundance in laser-treated groups for metabolites associated with phenylpropanoid biosynthesis (L-phenylalanine, trans-cinnamic acid, L-tyrosine, chlorogenic acid, coniferyl alcohol), carbon fixation in photosynthetic organisms (sedoheptulose 1,7-bisphosphate), photorespiration (L-glutamate), glutathione metabolism (L-glutamate, dehydroascorbic acid), flavonoid biosynthesis (hesperetin, chlorogenic acid, pelargonidin chloride, delphinidin), indole alkaloid biosynthesis (dialdehyde), and starch and sucrose metabolism (CDP-glucose, sucrose). Importantly, phenylalanine metabolism, glutathione metabolism, and starch and sucrose metabolism pathways exhibited active responses related to energy homeostasis under salt stress. Conversely, metabolites showing decreased accumulation in L15 versus L0 included those associated with anthocyanin biosynthesis (malvidin-3-(p-coumaroyl)-rutinoside-5-glucoside, delphinidin 3-glucoside 5-(caffeoyl-glucoside)), D-amino acid metabolism (D-lysine, L-arginine, L-aspartic acid), and pentose phosphate pathway (2-dehydro-3-deoxy-D-gluconate, α-D-ribose 1-phosphate) (**Figure 7E**).

### 3.7 Integrated transcriptomic and metabolomic analysis

**Figure 8:**
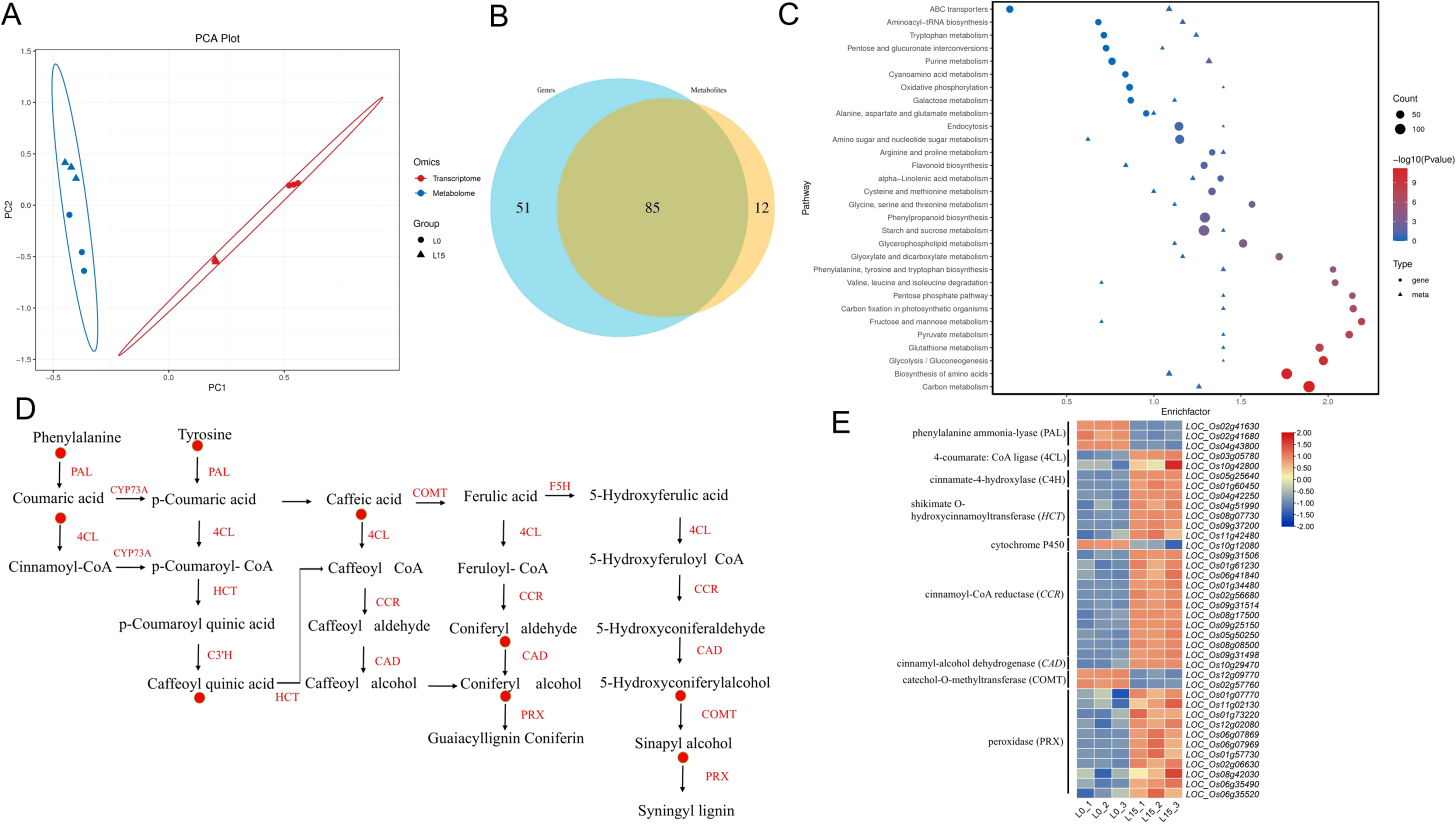
Correlation analysis of transcriptomic and metabolomic data. **(A)** Principal Component Analysis (PCA) plot from the integrated analysis. Sample points are denoted by shape (circles and triangles) representing different experimental groups. Transcriptomic samples are depicted in red, while metabolomic samples are shown in blue. **(B)** Venn diagram of pathways containing DEGs and DAMs. The blue circle represents transcriptomic (DEG) pathways, and the yellow circle represents metabolomic (DAM) pathways. **(C)** KEGG enrichment bubble plot. **(D)** Expression patterns of key DAMs in the phenylpropanoid biosynthetic pathway. The red circle graphs visually indicate differential metabolite up-regulation of expression. **(E)** Expression patterns of key DEGs in the phenylpropanoid biosynthetic pathway. Red color indicates up-regulation; blue color indicates down-regulation.

To investigate the intricate interplay between DEGs and DAMs in laser-treated rice seedlings under salt stress, we performed a comprehensive co-expression network analysis. PCA revealed significant differences in both transcript and metabolome (**Figure 8A**). Notably, metabolite module-gene module correlation chord diagrams confirmed strong positive correlations between numerous DEGs and DAMs (**Figure 8B**). Integrated analysis of DEGs and DAMs identified 83 co-regulated pathways shared between the transcriptomic and metabolomic datasets (**Figure 8B**). Specifically, KEGG analysis of the common pathways revealed that differences were significantly enriched in the pathways of carbon fixation, starch and sucrose metabolism, porphyrin and chlorophyll metabolism, phenylpropanoid biosynthesis, carbon metabolism and photosynthetic organisms, glutathione metabolism, and flavonoid biosynthesis (**Figure 8C**). Light affects the accumulation of phenylpropanoid compounds in plants, which play a role in salt tolerance in rice. Key metabolites in the phenylpropane metabolic pathway, coumaric acid, tyrosine, caffeic acid, 5-Hydroxyconiferylalcohol and sinapyl alcohol, accumulated more in L15 (**Figure 8D**). The expression patterns of key genes in the phenylalanine metabolic pathway, including phenylalanine/tyrosine ammonia-lyase (*OsPTAL*), 4-coumarate: CoA ligase (*Os4CL*), cinnamoyl-CoA reductase (*OsCCR*), shikimate O-hydroxycinnamoyltransferase (*OsHCT*), catechol-O-methyltransferase (*OsCOMT*) and cinnamyl-alcohol dehydrogenase (*OsCAD*), exhibited coordinated trends with the abundance of corresponding metabolites (**Figure 8E**).

## 4 Discussion

Light is a critical energy source for plant photosynthesis and an essential environmental signal in regulating growth, development, and stress responses (Leister, 2023; Li et al., 2025). Soil salinization, a major abiotic stress, significantly impairs plant growth and agricultural output (Singh, 2022). Previous research has shown that LDs outperform LEDs with similar peak wavelengths in enhancing photosynthesis and plant productivity (Okla et al., 2021; Al-Quraan et al., 2022). Unlike LEDs, LDs emit narrow-bandwidth, single-wavelength coherent light, offering precision for targeted photobiological manipulation (Li et al., 2025). However, the specific effects of LD emission peaks and spectral precision on stress resistance remain underexplored. This study investigates the physiological and molecular mechanisms by which LD light quality enhances salt tolerance in rice, establishing the foundation for its application in crop stress management. The interaction between laser light and plants depends on wavelength, exposure time, and intensity. Light quality, intensity, and photoperiod critically influence plant growth, development, and stress tolerance, with light quality exerting particularly complex effects (Bian et al., 2015; Roeber et al., 2021). Rice seedlings treated with red-blue LD (3:1 ratio) at 5, 10, and 15 μmol m^−2^ s^−1^ PPFD exhibited increased height, thicker stems, and stronger roots as intensity increased (**Figure 1**). These results align with prior studies showing that low-intensity laser treatment promotes root and shoot growth in rice, leading to increased tiller numbers, effective panicle formation, and improved grain yield (Qi et al., 2025).

Salt stress reduces rice yield by inhibiting biomass accumulation, often linked to decreased chlorophyll content and photosynthetic rate (Zheng et al., 2023). In this study, laser-treated rice seedlings (L5, L10, L15) showed higher chlorophyll a, b, and carotenoid content compared to controls, indicating that laser pretreatment enhances photosynthetic pigment synthesis to mitigate salt stress effects (**Figure 2**). Photosynthetic efficiency is a key determinant of plant resilience to abiotic stress (Miglani et al., 2021). Previous research has shown that adjusting the red-to-far-red light ratio enhances salt tolerance in cucumber by improving leaf photosynthetic capacity (Miao et al., 2023). Similarly, blue light promotes photosynthesis, stomatal opening, and transpiration under salinity, while supplementing red light with blue light enhances CO_2_ assimilation (Shimazaki et al., 2007). Here, red-blue LD irradiation significantly increased stomatal aperture, photosynthetic rate, and efficiency in rice leaves under salt stress (**Figure 3**). Similarly, He-Ne laser seed pre-treatment effectively enhances salinity stress tolerance in Ashwagandha (*Withania somnifera* (L.) Dunal) by regulating leaf gas exchange parameters and photosynthetic pigment contents, and increasing withanolide production (Thorat et al., 2024).

Salinity stress induces ion toxicity, nutrient imbalance, osmotic stress, and oxidative stress in plants (Arif et al., 2020). Plants mitigate these effects through Na^+^ exclusion, cytosolic K^+^ homeostasis, osmotic adjustment, and antioxidant defenses (Mohanty et al., 2023). Here, salt stress increased Na^+^ accumulation and K^+^ loss in rice roots and leaves, consistent with prior findings (**Figure 4**). Light signals, particularly red and blue light, modulate potassium uptake and stress tolerance. Laser treatment significantly reduced Na^+^ content, increased K^+^ content, and lowered the Na^+^/K^+^ ratio in roots and leaves compared to controls. Although salinity reduced K^+^ content, laser treatment mitigated this decline (**Figure 4**). Salt stress also inhibits uptake of essential minerals (N, P, Mg²^+^, Mn, Zn), critical for photosynthesis and metabolism. Laser treatment partially restored mineral absorption in salt-stressed seedlings (**Supplementary Figure S2**). This restorative effect may stem from the improvement of overall physiological status by LD and the potential modulation of specific nutrient uptake translocation pathways by light signaling (**Figure 1F**). Likewise, laser pre-sowing treatment of Moringa (*Moringa oleifera*) seeds significantly enhanced nutrient contents, with higher mineral (Fe, Mn, Ca, Cu, Zn) and biochemical (nitrogen, protein) levels in roots, shoots, and leaves compared to controls (Shafique et al., 2017). Salt stress also impairs photosynthesis, leading to excess energy in chloroplasts that causes ROS production (Ahanger et al., 2017; Wang et al., 2024). Plants enhance antioxidant capacity to maintain ROS homeostasis (Dumanović et al., 2021). Accordingly, H_2_O_2_ and MDA levels were significantly reduced in LD-treated seedlings (**Figures 5I–N**), confirming the effective alleviation of lipid peroxidation damage. Proline, an osmoprotectant and ROS scavenger, was significantly higher in laser-treated groups, improving cellular homeostasis and salt tolerance (Amir et al., 2024). In this study, we observed a significantly higher increase in proline content in leaves and roots as compared with control group (**Figures 5O, P**). These findings demonstrate that red-blue LD enhances salt tolerance by activating antioxidant systems, increasing proline accumulation, and reducing H_2_O_2_ and MDA levels. In another study, laser and magnetic field pre-sowing treatments significantly enhanced antioxidant enzyme activities (CAT, SOD, POD) and effectively ROS species in soybean seedlings, which is consistent with our study (Asghar et al., 2016).

Transcriptome enrichment analysis revealed that the differentially expressed genes (DEGs) were involved in pathways including photosynthesis, porphyrin and chlorophyll metabolism, ion transport, and phenylpropanoid biosynthesis and metabolism (**Figure 6**). Photosynthesis plays a critical role in maintaining energy supply and is therefore indispensable for plant resistance to stress. The chlorophyll a/b-binding (Lhc) superfamily proteins LHC a and LHC b are captured to form giant complexes (PSI-LHC a and PSII-LHC b) with PSI and PSII in cystoid membranes and are involved in photoprotection as well as responding to salt stress by stimulating the regulation and supply of light energy through bound chlorophyll a and b (Gao et al., 2018). Previous studies have indicated that psbS and other LHC-like proteins act as enhancers of photosynthesis and viability during abiotic stress, and their overexpression may improve crop yield and salt stress tolerance (Didaran et al., 2024). In this study, *psbS*, *Lhca*, and *Lhcb* were significantly up-regulated in the L5, L10, and L15 groups relative to L0, consist with chlorophyll content, which further confirming that LD modulates chlorophyll biosynthesis and signaling integration to improve photosynthetic efficiency of rice seedlings under salt stress (**Supplementary Figures S4A, S5A**). Most Lhc and PSI/PSII-associated genes are regulated by phytochrome (Leschevin et al., 2024). The differential expression of photoreceptor-related genes (*OsCRY1/2/3*, *OsPhyA/B/C*, *OsPIL12/13/14*) observed here further confirms the involvement of phytochrome and cryptochrome signaling pathways in regulation in rice salt stress response (**Supplementary Figures S4B, S5A**). It has been shown that down-regulation of *PhyB* can lead to reduced chloroplast damage, increased photosystem II efficiency, and activation of abiotic stress tolerance mechanisms in rice plants (Ganesan et al., 2017). Genes regulating ion transport and exclusion, such as SOSs, NHXs, and HKTs, contribute to mitigating cellular ion toxicity and play a vital role in maintaining intracellular ion homeostasis under high salinity (Lian et al., 2023; Bui et al., 2025). Previous studies have demonstrated that expression of OsNHX1 confers resistance to salt stress in rice (Farooq et al., 2021). Consistently, the expression of *OsNHX1* increased 4- to 6-fold in L10 and L15 under salt stress relative to L0, which further confirms the possible involvement of LD in Na/K ion homeostasis playing a positive regulatory role in rice salt tolerance (**Supplementary Figures S4C, S5B**). Treatment of tomato with red-blue light (3:1 ratio, R3B1) upregulated the expression levels of K^+^ transporter genes, leading to enhanced K^+^ uptake in root (Wang et al., 2021). It is hypothesized that red-blue LD could promote intracellular Na^+^ scavenging and enhances K^+^ uptake, thereby alleviating salt damage and maintaining salt tolerance in rice seedlings (**Supplementary Figure S4C**). In this study, LD pretreatment significantly increased the activities of SOD, POD, CAT, and APX, suggesting that LD stimulated the oxidative stress defense system in rice (**Figures 5A–H**). This activation was likely triggered by light signalling upon perception, resulting in up-regulation of the expression of key antioxidant enzyme genes (*OsSODC*, *OsPER*, *OsAPX*, *OsCAT*, which enhanced ROS scavenging and attenuated oxidative damage (**Supplementary Figures S4D, S5C**). This indicates that red-blue LD significantly increases antioxidant enzyme activity and osmolyte metabolism, leading to reduced salt-induced oxidative stress and consequently enhanced seedling stress resistance.

Plant growth is typically restricted under stress, while secondary metabolites often accumulate to provide protection (Saini et al., 2024). Metabolome analysis revealed that the differential metabolites were highly enriched in pathways including phenylalanine biosynthesis and metabolism, glutathione metabolism, flavonoid biosynthesis, and phenylpropanoid biosynthesis (**Figure 7D**). The significant enrichment of the glutathione metabolism pathway suggests that rice seedlings under salt stress may clear reactive ROS via reduced glutathione (GSH)-mediated pathways. Furthermore, flavonoids act as antioxidants, reducing ROS accumulation caused by salt stress and thereby minimizing oxidative damage (Hasanuzzaman et al., 2021). Under salt stress, LD can modulate flavonoid metabolism to decrease ROS levels and improve rice seedling salt tolerance. Phenylpropanoid metabolism is one of the most important metabolic pathways in plants. The pathway is initiated by phenylalanine, converted into cinnamate by PAL, C4H coverts cinnamate into p-coumarate, and p-coumarate is then activated by 4CL to form p-coumaroyl CoA. This leads to downstream specific synthesis routes for various phenylpropanoid metabolites, including lignans, flavonoids, coumarins, terpenoids, and anthocyanins, which play crucial roles in plant stress resistance (Yao et al., 2021). Combined transcriptomic and metabolomic analysis revealed the involvement of the phenylpropanoid biosynthesis pathway in the salt tolerance of *Sophora alopecuroides*, showing significant changes in the expression of genes and metabolites related to lignin and flavonoid synthesis. This suggests lignin and flavonoids participate in ROS scavenging and alleviating salt-induced damage (Zhu et al., 2021). In this study, integrated transcriptomic and metabolomic analysis showed a significant upregulation of DEGs and DAMs within the phenylpropanoid biosynthesis pathway in L15 (**Figure 8E**). In summary, under high salinity stress, LD enhances salt tolerance in rice seedlings through secondary metabolites generated via the glutathione metabolism, flavonoid biosynthesis, and phenylpropanoid biosynthesis pathways. Taken together, red-blue LD irradiation at a 3:1 ratio and 5–15 μmol m^−2^ s^−1^ PPFD enhances salt tolerance in rice seedlings by improving photosynthetic efficiency, ion homeostasis, and antioxidant capacity. These physiological improvements are driven by transcriptional and metabolic reprogramming, including upregulation of photosynthesis, ion transport, and antioxidant-related genes. This study provides reference parameters for supplementary lighting in the development of intelligent control systems, enabling dynamic adjustment of red-blue light ratios, light intensity, and exposure duration based on crop growth stages, real-time environmental conditions, and soil salinity. The combination of LD with intelligent control systems in plant factories can have significant practical application potential in agriculture. This study provides a foundation for applying LD technology to enhance crop resilience to abiotic stresses, offering a sustainable approach to improving agricultural productivity in saline environments.

## 5 Conclusions

LD pretreatment improved plant growth, photosynthetic efficiency, ion homeostasis, and antioxidant enzyme activity under salt stress. These effects were particularly evident at a 3:1 red-to-blue ratio and 10-15 μmol m^−2^ s^−1^ PPFD intensity. Transcriptomic analyses revealed upregulation of genes involved in photosynthesis, ion transport, and ROS scavenging, while metabolomic profiling identified increased accumulation of protective metabolites such as flavonoids, proline, and glutathione derivatives. This comprehensive study showed that red-blue LD irradiation significantly enhances salt tolerance in rice seedlings through coordinated physiological, transcriptomic, and metabolomic responses (**Supplementary Figure S6**). These findings establish a scientific foundation for the broader application of LD technology in precision agriculture. Given that LD has obvious advantages over LED light in terms of indoor horticultural plant growth, we can use LD in protected horticulture (greenhouses, plant factories) and optimize LD parameters to improve the yield and salt tolerance of these crops.

## Data Availability

The datasets presented in this study can be found in online repositories. The names of the repository/repositories and accession number(s) can be found below: https://www.ncbi.nlm.nih.gov/, PRJNA1274130.
