# Supplementary material for: Laser diode irradiation mitigates salt stress in rice through coordinated physiological and molecular responses
Source: Front Plant Sci. 2025 Sep 10;16:1653494. doi: 10.3389/fpls.2025.1653494 (PMC12457170; doi:10.3389/fpls.2025.1653494)
Supplement: Supplementary file 1 [file DataSheet1.docx]

Supplementary Material

Table S1 Sequences of primers used in the study

| Primer ID | Forward primer | Reverse Primer |
| --- | --- | --- |
| OsPsbR2 | GCGAATCCGAGCCATCCA | ACGCGTCAACACCGTCTT |
| OsPsbS2 | GCTTCGAGGGAAAGGGACA | TCCTCCAGCAGGCCCATA |
| OsLhca1 | CTGCCGACGATCCTGGTC | CCGCCGGGGTACTTCTTC |
| OsLhca2 | GGCTCGCCGGAGAAGATC | GAGGTTGTCGATGGGGCC |
| OsLhcb3 | GTCGAGGGCTACCGCATC | TTGAGCTCGGCGAAGGTG |
| OsLhcb4 | TCTCCGTCGAGTGGCTCA | AGATCGAGAACGGCAGCG |
| OsCRY1 | CCACGCGGCTCTTCTTCA | TCGACAACTTCCCACGGC |
| OsCRY2 | TGGTGGCTCAAGCAGTCG | AGGGTACTCTCGGCTCGG |
| OsPhyB | GAGTCGGTGTCCAAGGCC | CGATCTGCTGCTCGGAGG |
| OsPhyC | AGCTCCCGGGCAAGGATA | GCTTGCTGCTCAGGTCCA |
| OsSOS1 | GTGCCTTCGAGGTCGTCC | GGCAGAGAAGCACCAGCA |
| OsSOS2 | GGTGGGGAGGTACGAGGT | CGAGCACCTTCATGGCGA |
| OsSOS3 | CGAAGCAGTTCAAGCGGC | TGTTGTACAGCTCGCGCA |
| OsNHX1 | CCGACCCACACTGTCCAC | GGGAGAAGGGCACGAACC |
| OsNHX2 | GGACTACGGCTCGATCGC | CCCGATGATTAGCGCGGT |
| OsHAK2 | GCCGCTTGTGCTTGCTTT | CAAGGCTTGGACCCAGGG |
| OsHAK8 | CGAGCCTTGGCCTTGTCA | AACACCAGCGTCGAGTGG |
| OsHKT1 | ACGACCATGGTGGCAGTG | ACGACGTCCATGGGAGGA |
| OsHKT2 | CGGGGTGGTGGAGTGAAC | AGTTCGTGCGCTTGTGGA |
| OsSODC1 | GACACCGGATCTAGCGCC | GCCAACATTGAGGGCCCT |
| OsSODC2 | TGGCCGAGCTGTTGTTGT | GCCCCCAGCATTTCCAGT |
| OsPER1 | GGGCTGAAGGTCGGGTTC | TGGAAGTGCAGGCGGATG |
| OsPER2 | CAGATCACCCCGGCGTAC | GAAGCCCGGCATGAACCT |
| OsAPX1 | CTGCTGAGTGACCCTGCC | GCATCAGCGAACCCCAGT |
| OsAPX2 | ACCGAGCTTGTGAGTGGC | CAGTGGACGGAAGGCTGG |
| OsCAT2 | GCGGTGGGTTGATGCTCT | GAAGCCAGCTTCTGCCCA |
| OsCAT3 | GGACCTCGTCGGCAACAA | GGTTGGGCTTGAGCGAGT |
| UBI | AGGGTTCACAAGTCTGCCTA | TCTTCCATGCTGCTCTACCACA |

**
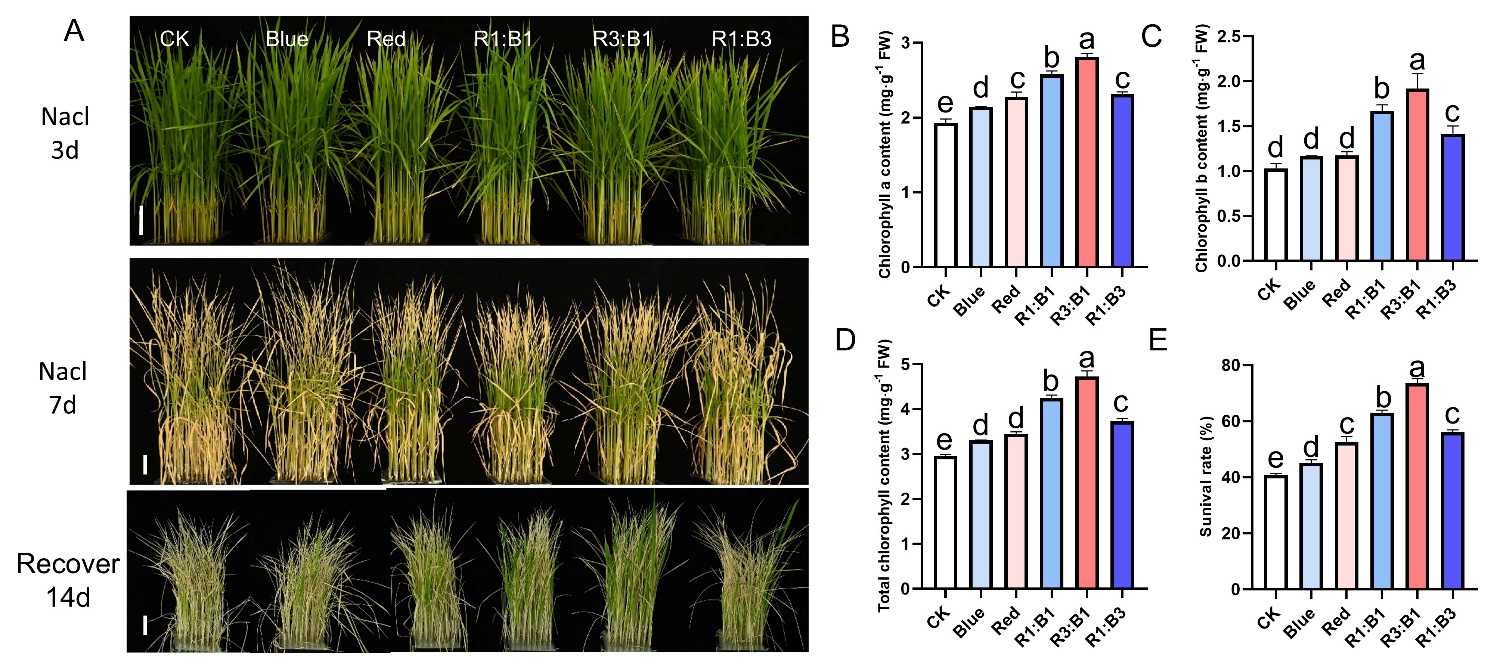
**

**Supplementary Figure 1.** Phenotypic and photosynthetic pigment analyses of rice seedlings under salt stress following LD irradiation with different spectral ratios. (A) Phenotypic images: Top to bottom - Control, 180 mM salt stress (7 days), and recovery phase (7 days); Left to right - Natural light (CK), blue LD (Blue), red LD (Red), red:blue 1:1 (R1B1), red:blue 3:1 (R3B1), and red:blue 1:3 (R1B3). (B-D) Photosynthetic pigment content determined at 3 days of salt stress: Chlorophyll a (B), Chlorophyll b (C), and Total chlorophyll (D). (E) Survival statistics after 14 days of recovery treatment. Different letters in each column indicate significant differences at P < 0.05, according to the Tukey–Kramer HSD test. The data are presented as the mean ± SE, n = 10.


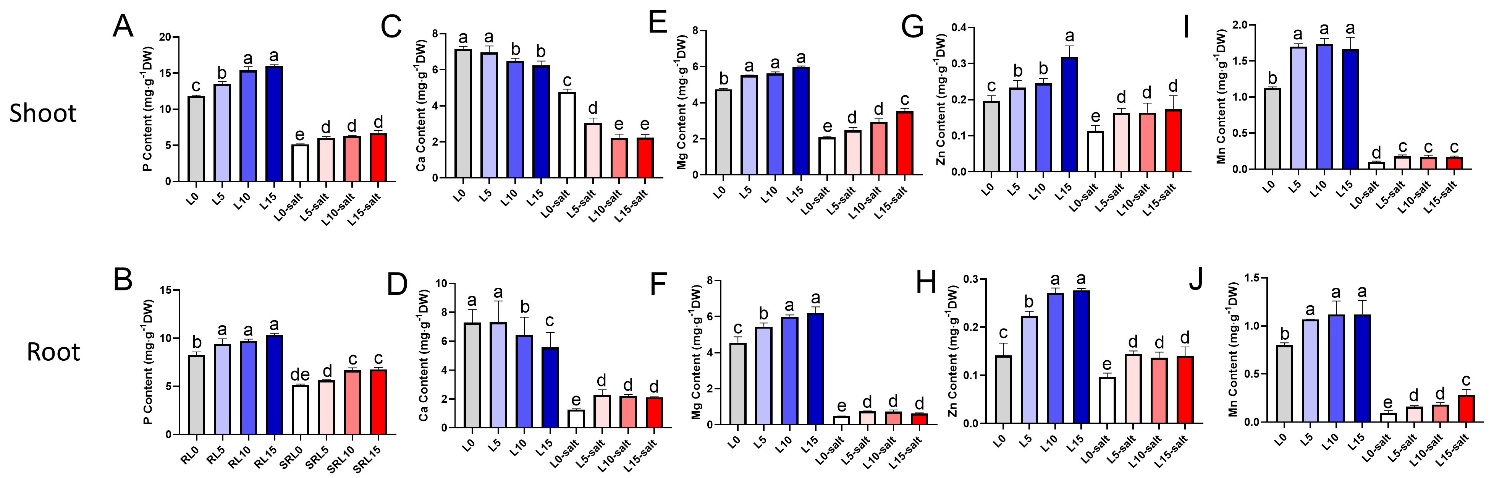


**Supplementary Figure 2.** Mineral ion contents in rice treated with different LD intensities under normal conditions and salt stress. (A, B) Phosphorus (P) content in leaves (A) and roots (B). (C, D) Calcium (Ca) content in leaves (C) and roots (D). (E, F) Magnesium (Mg) content in leaves (E) and roots (F). (G, H) Zinc (Zn) content in leaves (G) and roots (H). (I, J) Manganese (Mn) content in leaves (I) and roots (J).

**
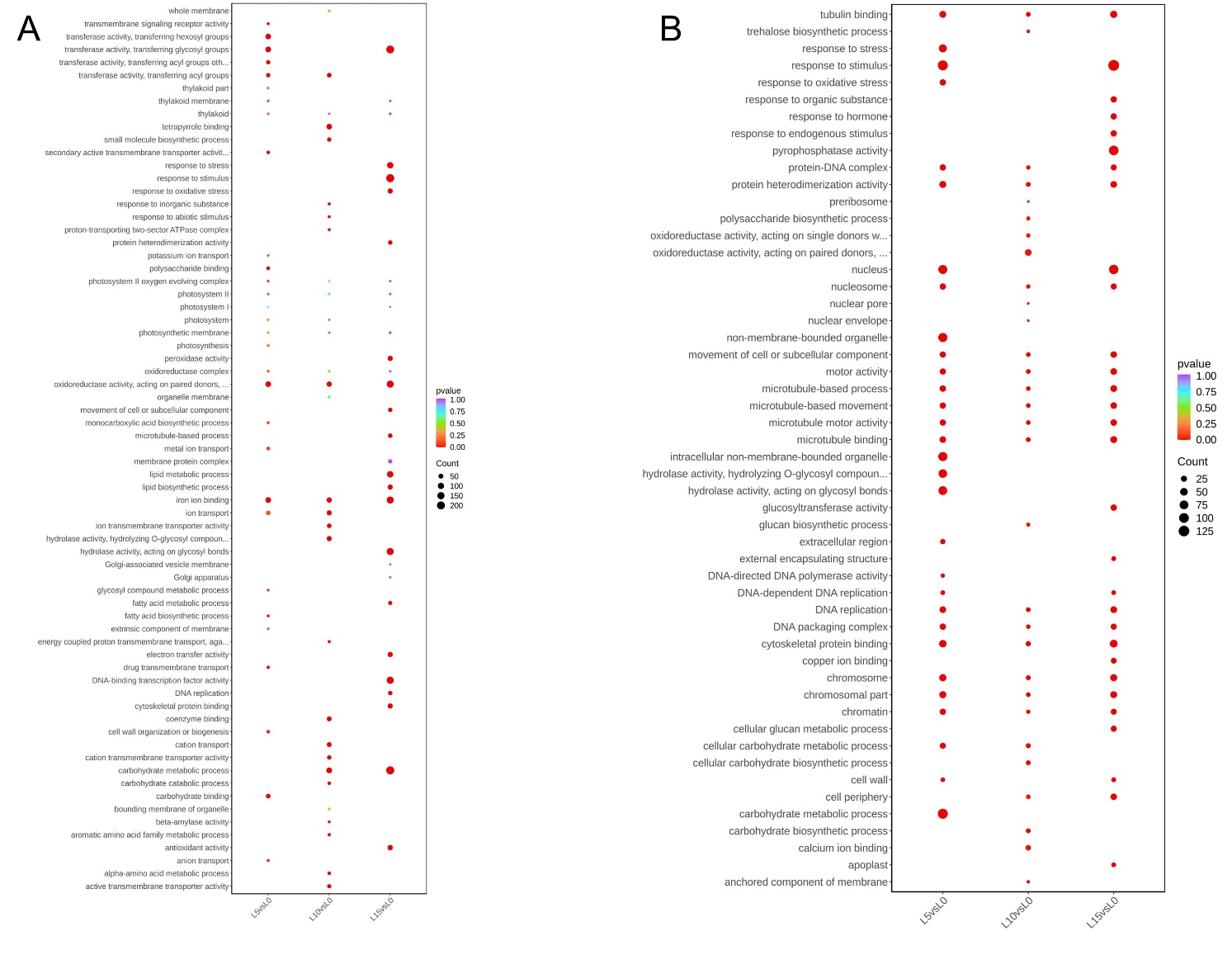
**

**Supplementary Figure 3.** GO analysis of DEGs in rice treated with different LD intensities until the three-leaf stage under salt stress. (A) GO enrichment of up-regulated DEGs across treatment groups. (B) GO enrichment of down-regulated DEGs across treatment groups.


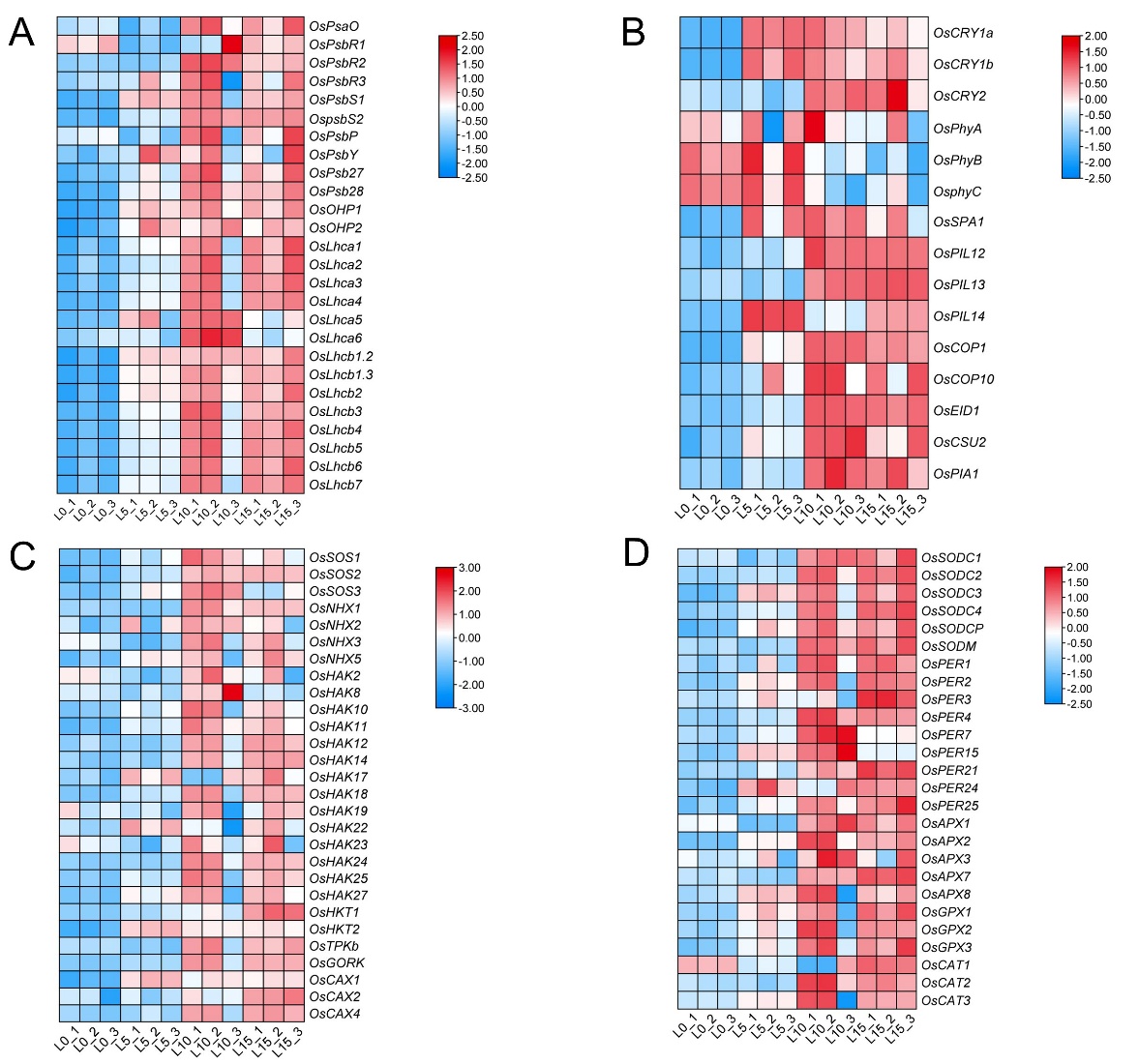


**Supplementary Figure 4.** Heatmap analysis of DEGs in rice treated with different laser intensities until the three-leaf stage under salt stress. (A) Heatmap of DEGs associated with photosynthesis pathways. (B) Heatmap of DEGs involved in light response pathways. (C) Heatmap of DEGs related to ionic homeostasis. (D) Heatmap of DEGs in antioxidant pathways.


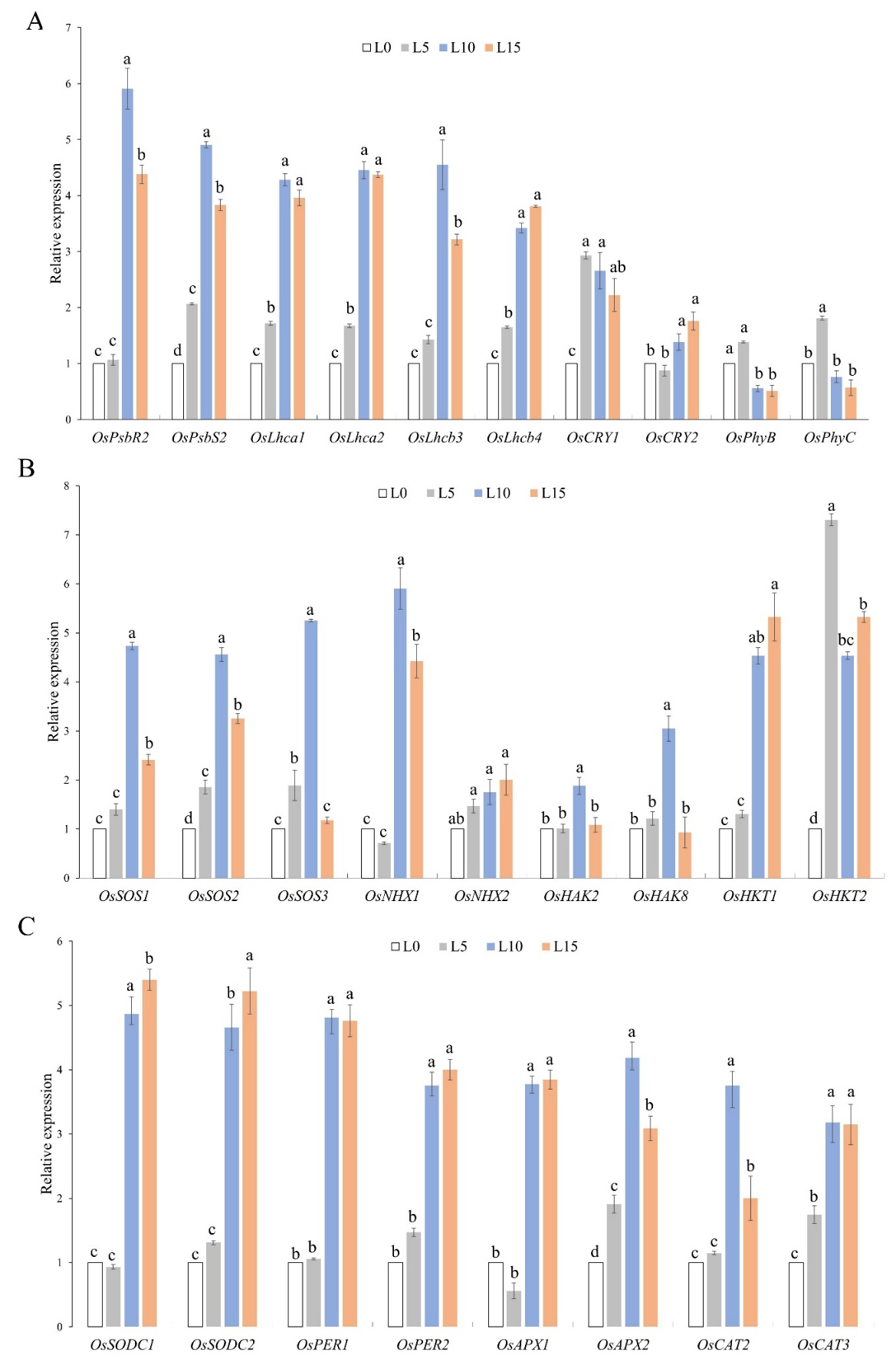


**Supplementary Figure 5.** QRT-PCR to verify the expression levels of key salt-responsive genes identified in the transcriptome analysis. The data were mean ± standard error (n = 3). anova was performed on the values between the four treatments under the same genotype. Different lowercase letters indicated differences at the P < 0.05 level.

**
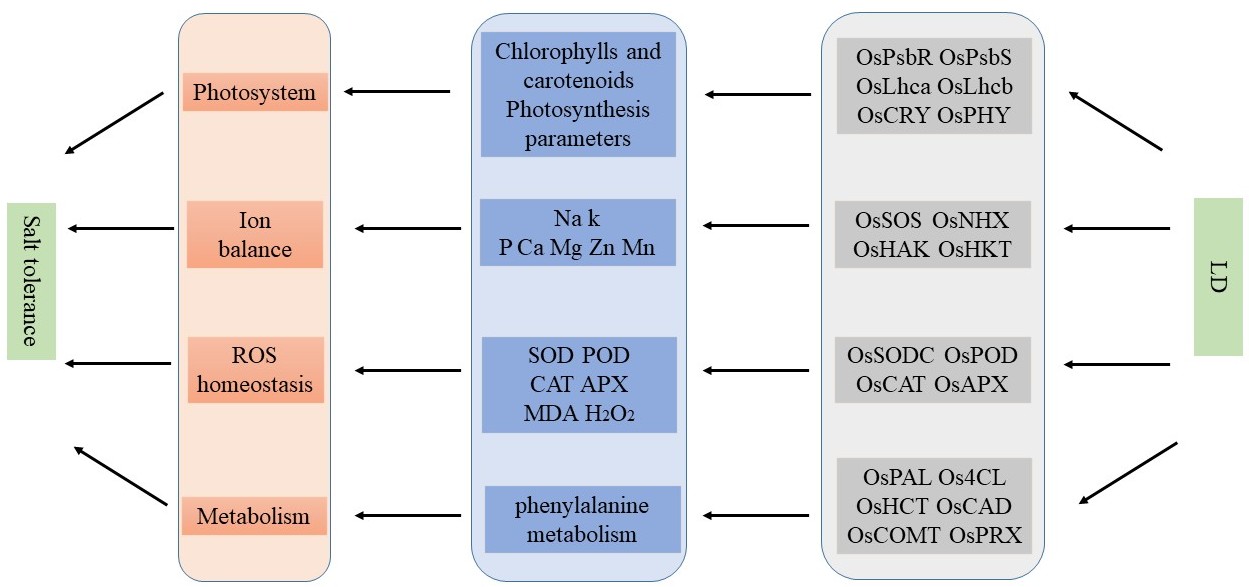
**

**Supplementary Figure 6.** Summarize the possible mechanisms by which LD mitigates salt stress.
